# Frailty and Outcomes Following Cardiopulmonary Resuscitation for Perioperative Cardiac Arrest

**DOI:** 10.1001/jamanetworkopen.2023.21465

**Published:** 2023-07-03

**Authors:** Matthew B. Allen, Ariela R. Orkaby, Samuel Justice, Daniel E. Hall, Frances Y. Hu, Zara Cooper, Rachelle E. Bernacki, Angela M. Bader

**Affiliations:** 1 Department of Anesthesiology, Perioperative and Pain Medicine, Brigham and Women’s Hospital, Harvard Medical School, Boston, Massachusetts; 2 New England Geriatric Research, Education, and Clinical Center (GRECC), Veterans Affairs Boston Healthcare System, Boston, Massachusetts; 3 Division of Aging, Brigham and Women’s Hospital, Harvard Medical School, Boston, Massachusetts; 4 Wolff Center, University of Pittsburgh Medical Center, Pittsburgh, Pennsylvania; 5 Center for Health Equity Research and Promotion, Veteran Affairs Pittsburgh Healthcare System, Pittsburgh, Pennsylvania; 6 GRECC, Veterans Affairs Pittsburgh Healthcare System, Pittsburgh, Pennsylvania; 7 Department of Surgery, University of Pittsburgh School of Medicine, Pittsburgh, Pennsylvania; 8 Department of Surgery, Brigham and Women’s Hospital, Harvard Medical School, Boston, Massachusetts; 9 Center for Surgery and Public Health, Brigham and Women’s Hospital, Harvard Medical School, Boston, Massachusetts; 10 Department of Psychosocial Oncology and Palliative Care, Dana-Farber Cancer Institute, Harvard Medical School, Boston, Massachusetts; 11 Division of Palliative Medicine, Department of Medicine, Brigham and Women’s Hospital, Harvard Medical School, Boston, Massachusetts

## Abstract

**Importance:**

Frailty is associated with mortality following surgery and cardiopulmonary resuscitation (CPR) for in-hospital cardiac arrest. Despite the growing focus on frailty as a basis for preoperative risk stratification and concern that CPR in patients with frailty may border on futility, the association between frailty and outcomes following perioperative CPR is unknown.

**Objective:**

To determine the association between frailty and outcomes following perioperative CPR.

**Design, Setting, and Participants:**

This longitudinal cohort study of patients used the American College of Surgeons National Surgical Quality Improvement Program, including more than 700 participating hospitals in the US, from January 1, 2015, through December 31, 2020. Follow-up duration was 30 days. Patients 50 years or older undergoing noncardiac surgery who received CPR on postoperative day 0 were included; patients were excluded if data required to determine frailty, establish outcome, or perform multivariable analyses were missing. Data were analyzed from September 1, 2022, through January 30, 2023.

**Exposures:**

Frailty defined as Risk Analysis Index (RAI) of 40 or greater vs less than 40.

**Outcomes and Measures:**

Thirty-day mortality and nonhome discharge.

**Results:**

Among the 3149 patients included in the analysis, the median age was 71 (IQR, 63-79) years, 1709 (55.9%) were men, and 2117 (69.2%) were White. Mean (SD) RAI was 37.73 (6.18), and 792 patients (25.9%) had an RAI of 40 or greater, of whom 534 (67.4%) died within 30 days of surgery. Multivariable logistic regression adjusting for race, American Society of Anesthesiologists physical status, sepsis, and emergency surgery demonstrated a positive association between frailty and mortality (adjusted odds ratio [AOR], 1.35 [95% CI, 1.11-1.65]; *P* = .003). Spline regression analysis demonstrated steadily increasing probability of mortality and nonhome discharge with increasing RAI above 37 and 36, respectively. Association between frailty and mortality following CPR varied by procedure urgency (AOR for nonemergent procedures, 1.55 [95% CI, 1.23-1.97]; AOR for emergent procedures, 0.97 [95% CI, 0.68-1.37]; *P* = .03 for interaction). An RAI of 40 or greater was associated with increased odds of nonhome discharge compared with an RAI of less than 40 (AOR, 1.85 [95% CI, 1.31-2.62]; *P* < .001).

**Conclusions and Relevance:**

The findings of this cohort study suggest that although roughly 1 in 3 patients with an RAI of 40 or greater survived at least 30 days following perioperative CPR, higher frailty burden was associated with increased mortality and greater risk of nonhome discharge among survivors. Identifying patients who are undergoing surgery and have frailty may inform primary prevention strategies, guide shared decision-making regarding perioperative CPR, and promote goal-concordant surgical care.

## Key Points

**Question:**

Is there an association between frailty and outcomes following cardiopulmonary resuscitation for perioperative cardiac arrest?

**Findings:**

In this cohort study of 3058 patients with perioperative cardiac arrests, frailty was associated with increased mortality and nonhome discharge. Among patients with severe frailty, increased Risk Analysis Index was associated with steadily increasing risk of mortality and nonhome discharge, and the association with mortality was most pronounced in the context of nonemergency surgery.

**Meaning:**

Identifying patients undergoing surgery with frailty may inform primary prevention strategies, guide shared decision-making regarding perioperative cardiopulmonary resuscitation, and promote goal-concordant surgical care.

## Teaser

This cohort study assesses the association between frailty, defined as a Risk Analysis Index of at least 40, and 30-day mortality and nonhome discharge following perioperative cardiopulmonary resuscitation (CPR).

## Introduction

The US health care system faces a surge in the number of older patients presenting for surgery.^1^ The American College of Surgeons (ACS) has focused attention on risk stratification, shared decision-making, and care tailored to age-specific vulnerabilities, including frailty.^2,3,4^ One particular risk older patients face is an increased risk of perioperative cardiac arrest,^5,6,7,8,9^ and practice guidelines advocate for preoperative discussion of preferences for cardiopulmonary resuscitation (CPR) in the perioperative period.^2,3,4^ Do-not-resuscitate orders are frequently reversed in this setting based in part on higher survival following perioperative resuscitation compared with CPR for in-hospital arrest (roughly 50% vs 25%).^10,11,12,13,14,15^

Frailty is a state of decreased physiological reserve predisposing to poor outcomes following stressors such as surgery.^16,17,18^ Frailty is associated with surgical complications and mortality^19,20,21,22,23,24,25,26,27^ and with increased risk of mortality following CPR for in-hospital cardiac arrest.^28,29,30,31^ Despite the growing focus on frailty as a basis for risk stratification^3,20,32,33^ and debate regarding the appropriateness of CPR in patients with frailty,^30,34^ the association between frailty and outcomes following perioperative CPR is unknown. This lack of evidence is a barrier to informed decision-making regarding CPR and goal-concordant management of complications of surgery and anesthesia.^35,36^

To provide an evidence base for decision-making regarding perioperative CPR in patients with frailty undergoing surgery, we used the ACS National Surgical Quality Improvement Program (NSQIP) database to identify patients who underwent CPR for perioperative cardiac arrest.^37^ Frailty was measured using the revised Risk Analysis Index (RAI), which has been widely studied and validated for use in surgical and nonsurgical patients.^38,39,40,41^ RAI is measured on a scale of 0 to 81, with 0 indicating no frailty and 81 indicating the most severe frailty. We determined the association between frailty defined as an RAI of at least 40 and outcomes following perioperative CPR. We hypothesized that frailty would be associated with increased odds of 30-day mortality and nonhome discharge.

## Methods

### Data Source

In this cohort study, we analyzed deidentified data from the ACS-NSQIP Participant Use Files from January 1, 2015, through December 31, 2020.^37^ The database includes cases from more than 700 participating US hospitals and incorporates more than 300 prospectively collected variables encompassing patient characteristics, procedure-related data, and 30-day outcomes. Analysis was performed from September 1, 2022, through January 30, 2023. This study adhered to the Strengthening the Reporting of Observational Studies in Epidemiology (STROBE) reporting guideline and was deemed exempt from review and informed consent by the Mass General Brigham Institutional Review Board owing to the use of deidentified data.

### Inclusion Criteria

We included patients 50 years or older who were undergoing noncardiac surgery and had CPR on postoperative day 0 (ie, intraoperatively or postoperatively on the day of surgery). The ACS-NSQIP^37^ defines CPR as “the absence of cardiac rhythm or presence of chaotic cardiac rhythm, intraoperatively or within 30 days following surgery, which results in a cardiac arrest requiring the initiation of CPR, which includes chest compressions.” Although the ACS-NSQIP records cardiac arrests beyond the day of surgery, we focused on perioperative arrests because outcomes among patients with frailty following in-hospital arrest have been previously reported,^28,29,30,31^ and outcome data are lacking for arrests in patients with frailty occurring in the arguably ideal setting of the operating room and closely monitored postoperative settings.^10^ Patients were excluded only if data were missing for 1 or more fields necessary to determine frailty, establish outcome, or perform multivariable analyses.

### Measurement of Frailty

Frailty was determined using the revised RAI for the primary analysis.^39^ The revised RAI is a recalibrated version of the administrative RAI, which was designed for use in patients undergoing surgery and incorporates comorbidities across multiple domains using variables from the ACS-NSQIP data set encompassing age, sex, functional status, dyspnea, weight loss, malignant neoplasms, and other comorbidities.^38,39^ Its development,^38^ validation,^39^ accuracy,^33^ feasibility,^33^ and clinical implementation^20^ have been well-described elsewhere. As previously reported, we addressed missing cognition data by scoring all patients as having no cognitive decline^40^ and scored patients as having cancer based solely on the variable for disseminated cancer (DISCANCER), as variables for chemotherapy (CHEMO) and radiotherapy (RADIO) are no longer collected.^41^ Although the RAI has previously been dichotomized across a cutoff of 30,^39^ we stratified frailty as RAI of less than 40 and 40 or greater because of the low number of arrests in patients with RAI of less than 30. We also performed a sensitivity analysis using an alternative tool for frailty measurement within the ACS-NSQIP that does not include age or sex: the 5-factor Modified Frailty Index (mFI-5), with 0 indicating no frailty and 5 indicating the most severe frailty.^42^ The mFI-5 can be calculated with only 5 variables,^42^ but has less fidelity to multidimensional character of frailty and is better understood as a comorbidity index than a measure of frailty.^17^

### Outcomes

The primary outcome was 30-day mortality following surgery and cardiac arrest. The secondary outcome was discharge to a destination other than home among patients who survived to hospital discharge.

### Statistical Analysis

Categorical demographic and comorbidity variables were compared between groups with frailty (RAI ≥40 and mFI-5 ≥2) and without frailty (RAI <40 and mFI-5 <2) using the χ^2^ test. The association between frailty and 30-day mortality was determined using univariable and multivariable logistic regression models. Univariable and multivariable logistic regression were likewise used to determine the association between frailty and nonhome discharge. The multivariable models controlled for race, emergency surgery, American Society of Anesthesiologists (ASA) physical status, and sepsis. These covariates were selected a priori based on clinical expertise and are known to be predictive factors associated with mortality following perioperative CPR.^5,6,7,8,9,10,13,14,15^ Consistent with prior analyses, covariates such as Charlson Comorbidity Index score, age, sex, and comorbidities were not included in the models to avoid collinearity.^22,25^ As a sensitivity analysis, we also constructed models using the mFI-5 that adjusted for age and sex in addition to the covariates included in the RAI models.^42^ Details on the mFI-5 cohort selection (eFigure in Supplement 1) and characteristics (eTable 1 in Supplement 1) as well as the results from the mFI-5 models (eTable 2 in Supplement 1) are found elsewhere.

To further investigate the association between frailty and outcomes after perioperative CPR, we built a multivariable logistic regression model of frailty analyzed as a continuous variable (RAI, 0-81) using restricted cubic splines fit via the rms package in R.^43^ Three knots were used for the spines, which were placed at the 10th, 50th, and 90th percentiles of the RAI in the cohort. We additionally investigated whether the association between frailty and mortality varied by urgency of the surgical procedure and Operative Stress Score (OSS) by testing for interactions between frailty (as a dichotomous variable) and these factors in separate multivariable logistic regression models.^24,44^

All analyses were prespecified. Results are reported as adjusted odds ratios (AORs) with 95% CIs. Results for the primary analyses were additionally reported as absolute risk differences with 95% CIs. The threshold for statistical significance was 2-sided *P* < .05, and all analyses were performed using R, version 4.1.2 (R Project for Statistical Computing).

## Results

**Figure 1.**
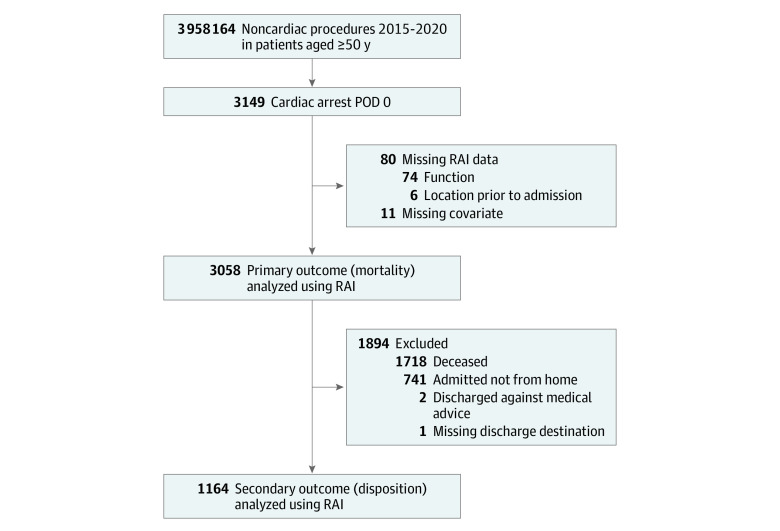
Study Flow Diagram Patients could be excluded from the study for more than 1 reason. POD indicates postoperative day; RAI, Risk Analysis Index.

There were 3 958 164 noncardiac surgical cases among patients 50 years or older in the ACS-NSQIP database from 2015 to 2020. Among these, there were 3149 reported cases of cardiac arrest on the day of surgery requiring CPR (incidence, 0.08% or 1 in 1250). After excluding 91 patients due to missing data, 3058 patients remained in the RAI cohort (Figure 1). Details regarding the mFI-5 cohort selection are presented in the eFigure in Supplement 1.

### Patient Characteristics

Patient characteristics for the RAI and mFI-5 cohorts are presented in Table 1 and eTable 1 in Supplement 1, respectively. The median age was 71 (IQR, 63-79) years; 1349 patients (44.1%) were women and 1709 (55.9%) were men. In terms of race, 377 patients (12.3%) were Black, 2117 (69.2%) were White, and 95 (3.1%) were other race (including American Indian or Alaska Native, Asian, and Native Hawaiian or other Pacific Islander). The RAI scores ranged from 14 to 71, the mean (SD) RAI was 37.73 (6.18), and 792 patients (25.9%) were considered frail (RAI ≥40). Patients with frailty were older (median age, 72 [IQR, 64-82] vs 70 [IQR, 62-78] years), less likely to be admitted from home (540 [68.2%] vs 1777 [78.4%]), and more likely to be partially (255 [32.2%] vs 11 [0.5%]) or totally dependent (65 [8.2%] vs 2 [0.1%]) than patients without frailty.

**Table 1.** Baseline Patient Characteristics Stratified by Risk Analysis Index

Characteristic	Patient group^a^			*P* value
	Overall (N = 3058)	RAI <40 (n = 2266)	RAI ≥40 (n = 792)	
Age, y				
Median (IQR)	71 (63-79)	70 (62-78)	72 (64-82)	NA
50-64	915 (29.9)	699 (30.8)	216 (27.3)	<.001^b^
65-74	988 (32.3)	762 (33.6)	226 (28.5)	
75-84	764 (25.0)	570 (25.2)	194 (24.5)	
≥85	391 (12.8)	235 (10.4)	156 (19.7)	
Sex				
Women	1349 (44.1)	987 (43.6)	362 (45.7)	.29
Men	1709 (55.9)	1279 (56.4)	430 (54.3)	
Race				
Black	377 (12.3)	217 (9.6)	160 (20.2)	<.001
White	2117 (69.2)	1593 (70.3)	524 (66.2)	
Other^c^	95 (3.1)	67 (3.0)	28 (3.5)	
Unknown	469 (15.3)	389 (17.2)	80 (10.1)	
Location prior to admission				
Home	2317 (75.8)	1777 (78.4)	540 (68.2)	<.001
Other	741 (24.2)	489 (21.6)	252 (31.8)	
Comorbidities and characteristics				
Functional status				
Independent	2725 (89.1)	2253 (99.4)	472 (59.6)	<.001
Partially dependent	266 (8.7)	11 (0.5)	255 (32.2)	
Totally dependent	67 (2.2)	2 (0.1)	65 (8.2)	
Congestive heart failure	244 (8.0)	28 (1.2)	216 (27.3)	<.001
Weight loss	130 (4.3)	11 (0.5)	119 (15.0)	<.001
Diabetes	418 (13.7)	201 (8.9)	217 (27.4)	<.001
Dyspnea	81 (2.6)	16 (0.7)	65 (8.2)	<.001
Kidney failure	351 (11.5)	10 (0.4)	341 (43.1)	<.001
Cancer	158 (5.2)	154 (6.8)	4 (0.5)	<.001
Preoperative sepsis				
None	2114 (69.1)	1685 (74.4)	429 (54.2)	<.001
Sepsis	212 (6.9)	122 (5.4)	90 (11.4)	
Septic shock	376 (12.3)	211 (9.3)	165 (20.8)	
SIRS	356 (11.6)	248 (10.9)	108 (13.6)	
ASA physical status				
1 and 2	294 (9.6)	278 (12.3)	16 (2.0)	<.001
3	1160 (37.9)	958 (42.3)	202 (25.5)	
4	1139 (37.2)	672 (29.7)	467 (59.0)	
5	465 (15.2)	358 (15.8)	107 (13.5)	
Emergency surgery	1195 (39.1)	862 (38.0)	333 (42.0)	.05
General anesthesia	2852 (93.3)	2112 (93.2)	740 (93.4)	.82
Elective surgery^d^	1248 (40.9)	1058 (46.8)	190 (24.0)	<.001
Operative Stress Score^e^				
1-2 (low)	496 (17.1)	359 (16.8)	137 (17.9)	<.001
3 (moderate)	1475 (50.8)	1026 (48.0)	449 (58.7)	
4-5 (high)	930 (32.1)	751 (35.2)	179 (23.4)	

Abbreviations: ASA, American Society of Anesthesiologists; NA, not applicable; RAI, Risk Analysis Index; SIRS, systemic inflammatory response syndrome.

^a^ Frailty was measured using the revised RAI (stratified as RAI <40 and RAI ≥40 [frailty]). Unless indicated otherwise, data are expressed as No. (%) of patients.

^b^ Corresponds to a χ^2^ test, as age was analyzed by category (the database censors ages for patients aged ≥90 years).

^c^ Includes American Indian or Alaska Native, Asian, and Native Hawaiian or other Pacific Islander.

^d^ Includes 3053 for elective surgery and nonelective surgery (5 [0.2%] missing).

^e^ Includes 2901 for Operative Stress Score low, moderate, and high (157 [5.1%] missing).

A total of 1195 arrests (39.1%) occurred in the context of emergency surgery, and 1971 of 2901 (67.9%) occurred in the setting of low- or moderate-stress procedures (ie, OSS 1-3). Patients with frailty were more likely to have their arrest in the context of emergency surgery (333 [42.0%] vs 862 [38.0%]) and in the setting of low- or moderate-stress procedures (586 of 765 [76.6%] vs 1385 of 2136 [64.8%]) compared with patients without frailty. Use of general anesthesia did not differ between patients with and without frailty (740 [93.4%] vs 2112 [93.2%]).

### Mortality

**Figure 2.**
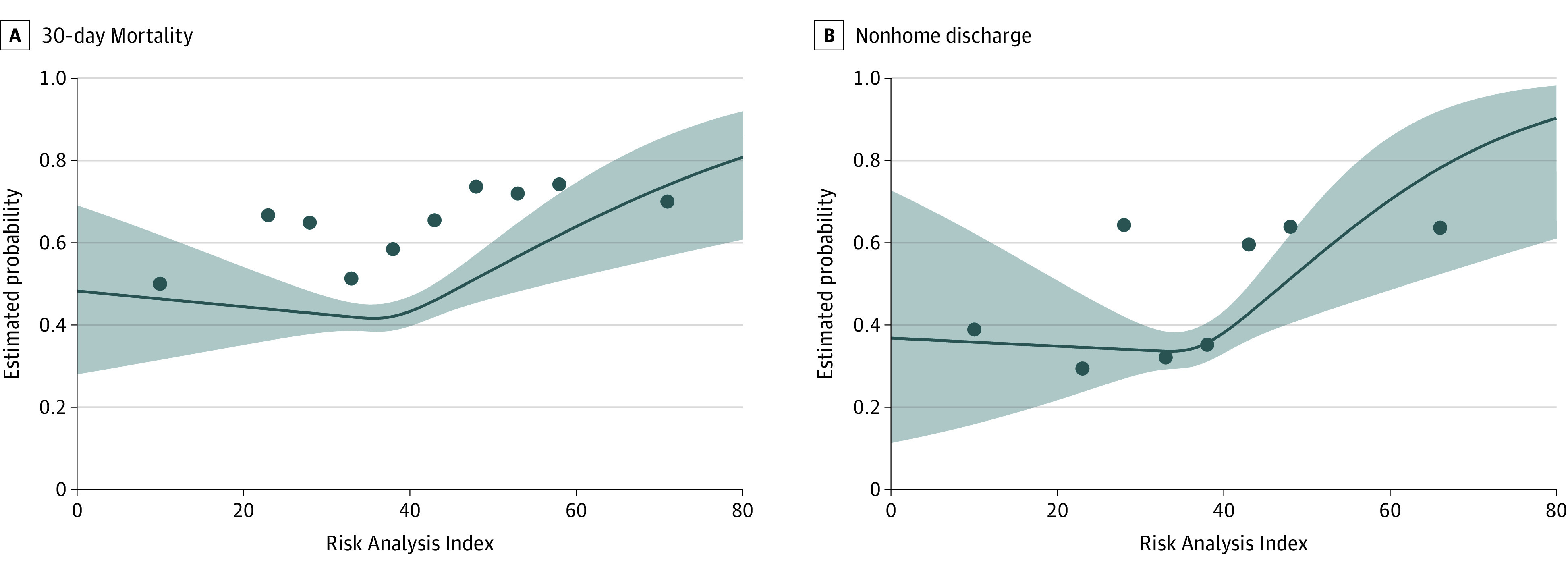
Multivariable Restricted Cubic Splines for the Estimated Probabilities of Outcomes Following Perioperative Cardiopulmonary Resuscitation by Risk Analysis Index Blue dots represent raw mortality and nonhome discharge rates for Risk Analysis Index (RAI) ranges; RAI bins were 0 to 20, 21 to 25, 26 to 30, 31 to 35, 36 to 40, 41 to 45, 46 to 50, 51 to 55, 56 to 60, and 61 to 81 for mortality and 0 to 20, 21 to 25, 26 to 30, 31 to 35, 36 to 40, 41 to 45, 46 to 50, and 51 to 81 for nonhome discharge. Larger bins were used at the lower and upper regions of the RAI due to sparse patient numbers in these ranges. Dots are plotted at the midpoint of each bin.

A total of 1793 patients (58.6%) died within 30 days after surgery (534 [67.4%] with frailty vs 1259 [55.6%] without frailty). Multivariable logistic regression adjusting for race, ASA physical status, sepsis, and emergency surgery demonstrated a positive association between frailty and mortality (AOR, 1.35 [95% CI, 1.11-1.65]; *P* = .003), corresponding to an absolute risk difference of 6% (0.06 [95% CI, 0.02-0.10]) (Table 2). Sensitivity analysis did not demonstrate an association between mFI-5 of at least 2 and mortality (eTable 2 in Supplement 1). Multivariable spline regression analysis demonstrated a nonlinear association between the RAI defined as a continuous variable and log odds of mortality (*P* = .02) with steadily increasing probability of mortality with increasing RAI above 37 (Figure 2).

**Table 2.** Logistic Regression Models Examining the Association Between Frailty and Outcomes Following Perioperative Cardiopulmonary Resuscitation and Absolute Risk Differences^a^

Outcome	No. of events/No. at risk		Odds ratio (95% CI)	*P* value	Adjusted odds ratio (95% CI)^b^	*P* value	Estimated probability^c^		Absolute risk difference (95% CI)
	RAI <40	RAI ≥40					RAI <40	RAI ≥40	
30-d Mortality	1259/2266	534/792	1.66 (1.40-1.96)^d^	<.001	1.35 (1.11-1.65)^d^	.003	0.57	0.63	0.06 (0.02-0.10)
Nonhome discharge	322/950	127/214	2.85 (2.10-3.87)^d^	<.001	1.85 (1.31-2.62)^d^	<.001	0.36	0.49	0.13 (0.05-0.21)

Abbreviation: RAI, Risk Analysis Index.

^a^ Perioperative arrests defined as arrests occurring intraoperatively or postoperatively on the day of surgery (ie, postoperative day 0).

^b^ Adjusted for American Society of Anesthesiologists physical status, race, emergency surgery, and sepsis.

^c^ Obtained by calculating the mean of the entire cohort and used to form the absolute risk differences; 95% CIs for the absolute risk differences were calculated based on bootstrapping with 1000 bootstrap replicates.

^d^ Indicates ratio of patients with RAI of 40 or greater vs less than 40.

**Figure 3.**
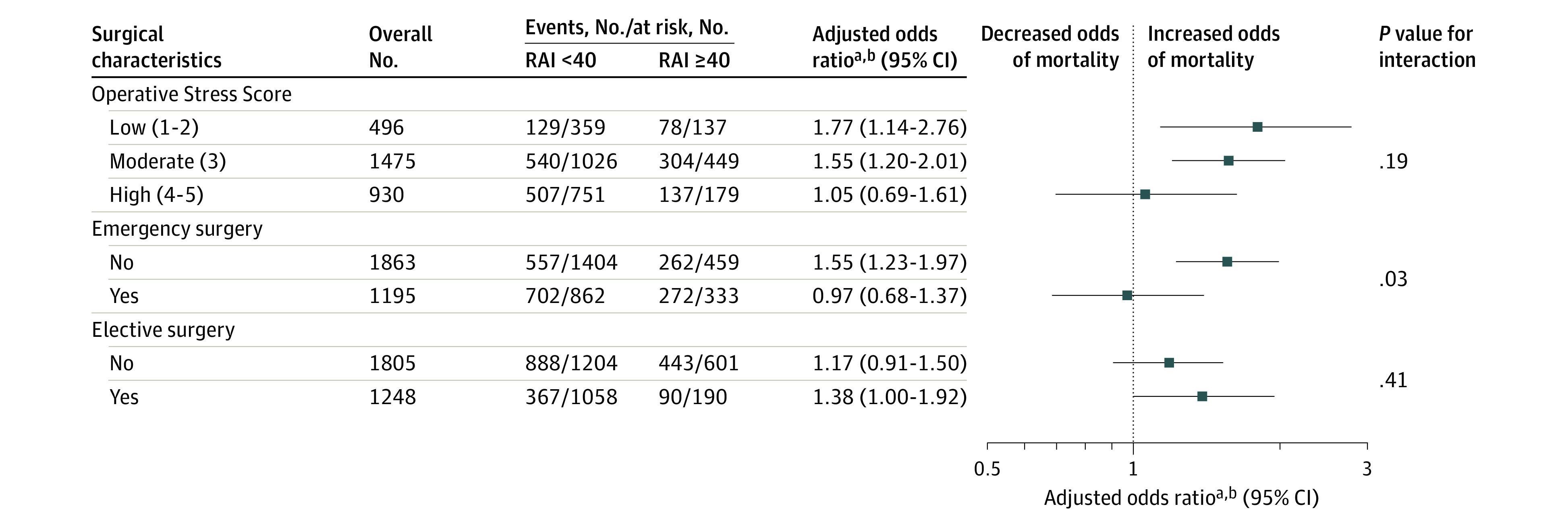
Adjusted Odds Ratios for Association Between Frailty and Mortality Following Perioperative Cardiopulmonary Resuscitation by Surgical Characteristics For this study, frailty was defined as a Risk Analysis Index (RAI) of 40 or greater. ^a^Ratio of patients with RAI of 40 or greater vs less than 40. ^b^Adjusted for American Society of Anesthesiologists physical status, race, emergency surgery, and sepsis.

The only significant interaction between frailty and surgical subgroup was for procedure urgency, with frailty demonstrating an increased risk of mortality following arrests in the setting of nonemergency surgery (AOR, 1.55 [95% CI, 1.23-1.97]) compared with emergent surgery (AOR, 0.97 [95% CI, 0.68-1.37]; *P* = .03 for interaction). There were no significant interactions when results were stratified by OSS or elective vs nonelective surgery (Figure 3).

### Nonhome Discharge

Among the 1164 patients who were admitted from home and survived to discharge, 449 (38.6%) were discharged to a destination other than home (127 [59.3%] with frailty vs 322 [33.9%] without frailty). Frailty was associated with increased odds of nonhome discharge after adjusting for ASA status, race, sepsis, and emergency surgery (AOR, 1.85 [95% CI, 1.31-2.62]; *P* < .001), corresponding to an absolute risk difference of 13% (AOR, 0.13 [95% CI, 0.05-0.21]) (Table 2). Sensitivity analysis using the mFI-5 generated similar results (eTable 2 in Supplement 1). Multivariable spline regression analysis demonstrated a nonlinear association between the RAI defined as a continuous variable and log odds of nonhome discharge (*P* = .048), with steadily increasing probability of nonhome discharge with increasing RAI above 36 (Figure 2).

## Discussion

In this longitudinal cohort study of patients 50 years and older from the ACS-NSQIP database who underwent perioperative CPR, frailty was associated with significantly increased odds of 30-day mortality and discharge to destinations other than home. Although roughly 1 in 3 patients with an RAI of 40 or greater survived at least 30 days following perioperative CPR, higher frailty burden was associated with increased mortality and greater risk of nonhome discharge among survivors. Our findings extend a growing body of literature defining the vulnerabilities of older patients undergoing surgery, highlight possible opportunities for improving outcomes, and provide a heretofore absent base of evidence to guide decision-making regarding CPR in patients with frailty who undergo surgery.

Survival following CPR for cardiac arrest in the perioperative setting (roughly 50%)^10,12,13,14,15^ is thought to be higher than for in-hospital arrests (25%)^11^ because of more favorable etiology (eg, reversible effects of anesthesia), immediate recognition and intervention, and resuscitation led by specialized clinicians with detailed knowledge of the patient.^10^ Growing evidence of poor outcomes among patients with frailty who undergo CPR for in-hospital arrest has stimulated debate regarding the appropriateness of CPR in patients with frailty and has raised concerns that it may border on futility.^28,29,30,31,34^ Multiple studies^30,45,46^ have demonstrated mortality in excess of 95% among patients with frailty following in-hospital CPR, and meta-analyses^28,29^ indicate that patients with frailty have roughly 3-fold increased odds of mortality compared with those without frailty. Cohorts studied thus far have been relatively small, with sample sizes ranging from 90 to 570 patients and a recent meta-analysis including a total of 1704 patients.^29^ Importantly, to our knowledge, none of the existing literature has focused on the association between frailty and outcomes following CPR for arrests in the perioperative setting. Although direct comparison with the current study is complicated by methodological and contextual differences, the weaker association between frailty and mortality in the ACS-NSQIP cohort compared with existing evidence suggests that results from other settings should be applied to perioperative decision-making with caution.

Most arrests occurred in the context of nonemergency surgery, and we found evidence of interaction between frailty and nonemergency surgery in association with increased mortality following perioperative CPR. This finding may reflect a tendency for patients with a high frailty burden and high acuity to undergo nonoperative management.^47^ Alternatively, the lack of association between frailty and outcomes following CPR in the setting of emergency surgery may indicate that clinical trajectories in this context are driven by factors other than frailty (eg, sepsis, shock).^14^ In addition, the association between frailty and mortality following arrest in the context of nonemergency surgery suggests that in many cases, there may be opportunities to optimize modifiable risk factors and formulate surgical and anesthesia plans tailored to patients’ vulnerabilities and preferences.^47,48,49,50,51,52^

Fewer than 1 in 4 arrests in patients with frailty occurred in the context of high-stress procedures (ie, OSS, 4-5), and we did not find an association between frailty and increased mortality following CPR in this subgroup. As with literature demonstrating stronger association between frailty and negative outcomes following nonemergency,^53^ ambulatory,^21^ and lower-risk surgery,^19,23,24,25,26^ the lack of association between frailty and mortality following arrests occurring in the setting of high-stress procedures may be attributable to meticulous patient selection. However, variable associations between frailty and mortality based on procedure characteristics may also reflect differences in management. For example, after implementing a facility-wide frailty screening initiative, Hall et al^20^ and Ernst et al^47^ observed a 19% reduced risk of 180-day mortality in patients with frailty undergoing surgery that was not attributable to changes in patient selection. Improved outcomes after identifying patients with frailty may instead reflect attention to patient-related risks as well as heightened vigilance promoting primary prevention, early recognition, and timely treatment of complications.^20^ The fact that most arrests in patients with frailty occurred in the context of low- and moderate-stress procedures supports the view that in patients with frailty, no procedure or anesthetic should be considered low risk and highlights the importance of universal screening as a basis for risk-mitigation strategies.

Our results should also inform decision-making regarding management of perioperative cardiac arrest in high-risk patients. The common practice of reversing existing do-not-resuscitate orders for surgery is based partially on lower mortality following perioperative arrest compared with in-hospital or out-of-hospital arrests.^10,11^ Though logical, this reasoning oversimplifies the risk profile of perioperative CPR and fails to consider outcomes other than mortality that seriously ill patients value.^35,36,54^ Our findings do not support viewing resuscitation as futile in patients with frailty who are undergoing surgery, but they do suggest that the appropriateness of CPR should not be universally assumed, particularly in patients with a high preoperative frailty burden. Instead, management of clinical decompensation in the perioperative period should be the subject of a shared decision-making process to establish a plan aligned with patients’ priorities whenever possible.^2,35,36^ These discussions frequently overlap with consideration of whether surgery itself is concordant with patients’ priorities and are likely enhanced by the early involvement of palliative care specialists and geriatricians.^47,48,49,50,51,52^ The data reported herein can help clinicians communicate the risks to patients by anticipating trajectories under the best, worst, and most likely scenarios.^55^

### Strengths and Limitations

This study has several strengths. To our knowledge, the cohort is the largest sample ever studied to evaluate the association between frailty and CPR outcomes in any setting and is one of the largest used to describe outcomes following perioperative CPR. We optimized internal validity by restricting the analysis to the cohort of interest (patients undergoing noncardiac surgery and aged ≥50 years with arrests on the day of surgery) while controlling for confounders and maintaining power to detect differences by subgroup. Our use of a national database with a contemporary sample from more than 700 hospitals and a well-validated frailty measure designed for use in patients undergoing surgery provides robust external validity.^37,38,39^ The lack of association between the mFI-5 and mortality following perioperative CPR supports concerns that comorbidity-centered indices fail to capture the multidimensional character of frailty and underlines the utility of the RAI as a basis for preoperative risk stratification.^17,56,57^

Our findings should be interpreted with attention to the study’s limitations. Frailty has no consensus definition, and although our findings reflect use of the most thoroughly validated NSQIP-based measure, they are subject to reinterpretation based on future analyses using other data sets and frailty indices.^33^ Because clinical trajectories of patients with and without frailty may continue to diverge for months following stressors,^24^ the 30-day follow-up period may have biased findings toward the null hypothesis. Outcomes vary by arrest etiology and other variables not captured in the database (eg, presence of a shockable rhythm, duration of resuscitation, intraoperative vs postoperative arrest),^11,12,13,14^ and we cannot exclude the possibility of residual confounding. Because the database does not include site-specific identifiers, we were likewise unable to evaluate the importance of site-specific differences in outcome.^37^ Finally, we were unable to evaluate functional trajectory and other patient-centered outcomes among survivors, which should be prioritized in future research.^54^

## Conclusions

In this cohort study, frailty was associated with increased odds of 30-day mortality and nonhome discharge following perioperative CPR. Although roughly 1 in 3 patients with an RAI of 40 or greater survived at least 30 days following perioperative CPR, higher frailty burden was associated with steadily increasing probabilities of mortality and nonhome discharge among survivors. Identifying patients with frailty prior to surgery may inform implementation of strategies aimed at preventing cardiac arrest, guide shared decision-making regarding perioperative CPR, and promote goal-concordant management of complications of surgery and anesthesia.

## Notes

Supplement 1. **eFigure.** Flow Diagram for 5-factor Modified Frailty Index Cohort Selection **eTable 1.** Baseline Characteristics Stratified by the 5-Factor Modified Frailty Index **eTable 2.** Univariable and Multivariable Logistic Regression Models Examining the Association Between Frailty and Outcomes Following Perioperative Cardiopulmonary Resuscitation

Supplement 2.
Data Sharing Statement
