# Supplementary material for: Frailty and Outcomes Following Cardiopulmonary Resuscitation for Perioperative Cardiac Arrest
Source: JAMA Netw Open. 2023 Jul 3;6(7):e2321465. doi: 10.1001/jamanetworkopen.2023.21465 (PMC10318473; doi:10.1001/jamanetworkopen.2023.21465)
Supplement: Supplement 1. — eFigure. Flow Diagram for 5-factor Modified Frailty Index Cohort Selection eTable 1. Baseline Characteristics Stratified by the 5-Factor Modified Frailty Index eTable 2. Univariable and Multivariable Logistic Regression Models Examining the Association Between Frailty and Outcomes Following Perioperative Cardiopulmonary Resuscitation [file jamanetwopen-e2321465-s001.pdf]

Supplementary Online Content

Allen MB, Orkaby AR, Justice S, et al. Frailty and outcomes following cardiopulmonary resuscitation for perioperative cardiac arrest. *JAMA Netw Open*. 2023;6(7):e2321465. doi:10.1001/jamanetworkopen.2023.21465

eFigure. Flow Diagram for 5-factor Modified Frailty Index Cohort Selection
eTable 1. Baseline Characteristics Stratified by the 5-Factor Modified Frailty Index
eTable 2. Univariable and Multivariable Logistic Regression Models Examining the Association Between Frailty and Outcomes Following Perioperative Cardiopulmonary Resuscitation

This supplementary material has been provided by the authors to give readers additional information about their work.

© 2023 Allen MB et al. *JAMA Network Open*.

eFigure. Flow Diagram for 5-factor Modified Frailty Index Cohort Selection

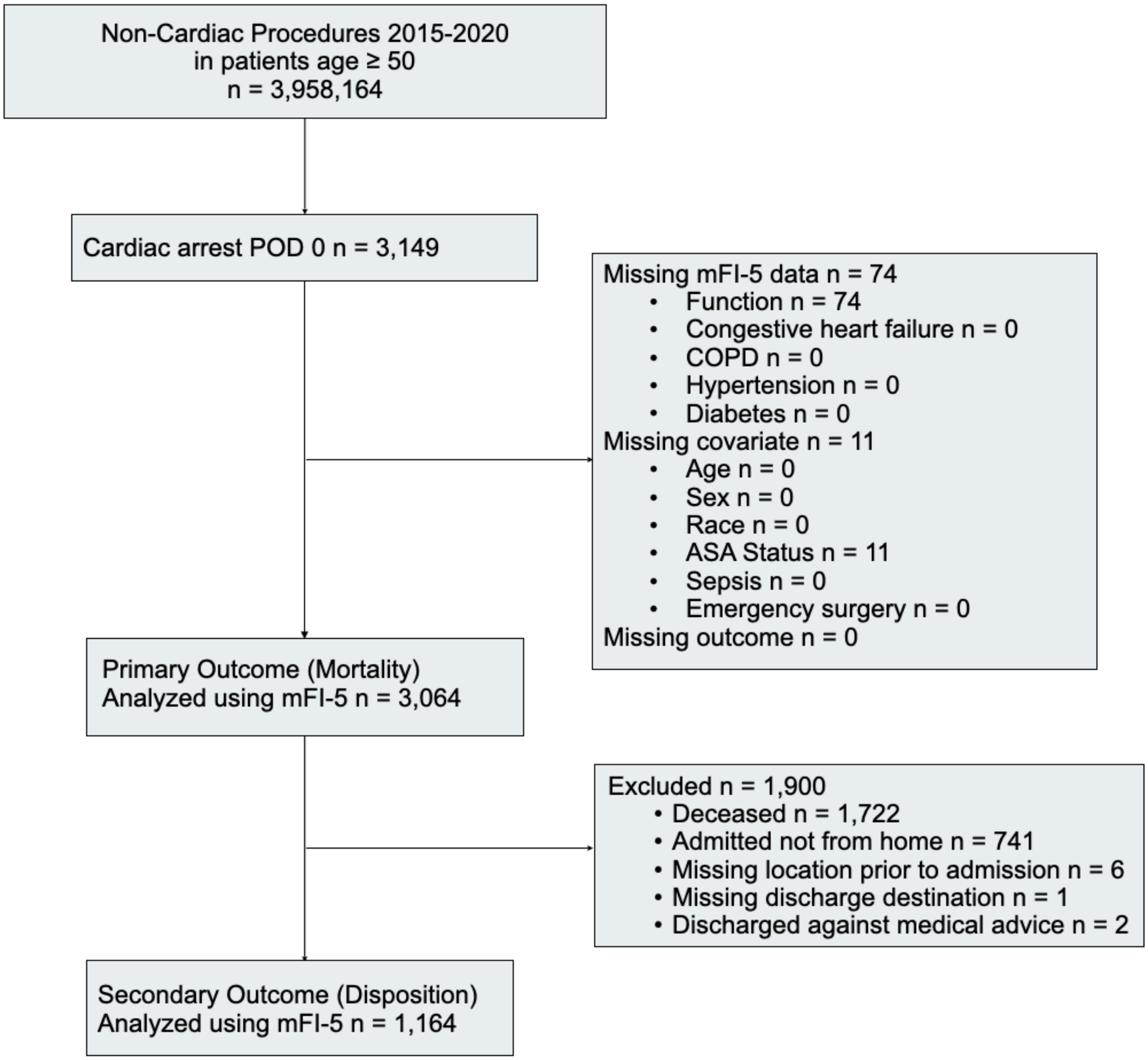

Abbreviations: mFI-5 = 5-factor Modified Frailty Index; ASA = American Society of Anesthesiologists; POD = postoperative day

© 2023 Allen MB et al. *JAMA Network Open*.

eTable 1. Baseline Characteristics Stratified by the 5-Factor Modified Frailty Index

Demographics	mFI-5 < 2<br>n (%)	mFI-5 ≥ 2<br>n (%)	*P*
Overall	2130	934	
Age, Median [IQR], y	70 [62, 78]	72 [64, 80]	
50-64	677 (31.8)	240 (25.7)	<0.001[a]
65-74	698 (32.8)	292 (31.3)	
75-84	518 (24.3)	248 (26.6)	
≥ 85	237 (11.1)	154 (16.5)	
Sex			
Female	909 (42.7)	442 (47.3)	0.02
Male	1221 (57.3)	492 (52.7)	
Race			
White	1471 (69.1)	649 (69.5)	<0.001
Black	230 (10.8)	148 (15.8)	
Other[b]	67 (3.1)	28 (3.0)	
Unknown	362 (17.0)	109 (11.7)	
Location prior to admission[c]			
Home	1646 (77.4)	671 (72.0)	0.001
Other	480 (22.6)	261 (28.0)	
**Comorbidities/Characteristics**	**mFI-5 < 2<br>n (%)**	**mFI-5 ≥ 2<br>n (%)**	***P***
Functional status			
Independent	2088 (98.0)	642 (68.7)	<0.001
Partially dependent	31 (1.5)	236 (25.3)	
Totally dependent	11 (0.5)	56 (6.0)	
Congestive Heart Failure	22 (1.0)	223 (23.9)	<0.001
Weight loss	78 (3.7)	52 (5.6)	0.02
COPD	81 (3.8)	376 (40.3)	<0.001
Diabetes	30 (1.4)	390 (41.8)	<0.001
Hypertension	1268 (59.5)	911 (97.5)	<0.001

Dyspnea	28 (1.3)	54 (5.8)	<0.001
Renal Failure	147 (6.9)	205 (21.9)	<0.001

Baseline Characteristics Stratified by the 5-factor Modified Frailty Index (Continued)

Comorbidities/Characteristics	mFI-5 < 2<br>n (%)	mFI-5 ≥ 2<br>n (%)	*P*
Cancer	124 (5.8)	34 (3.6)	0.01
Preoperative Sepsis			
None	1538 (72.2)	579 (62.0)	<0.001
SIRS	233 (10.9)	124 (13.3)	
Sepsis	123 (5.8)	89 (9.5)	
Septic Shock	236 (11.1)	142 (15.2)	
ASA Physical Status			
1 and 2	272 (12.8)	22 (2.4)	<0.001
3	857 (40.2)	304 (32.5)	
4	640 (30.0)	503 (53.9)	
5	361 (16.9)	105 (11.2)	
Emergency Surgery	847 (39.8)	352 (37.7)	0.28
General Anesthesia	1988 (93.3)	869 (93.0)	0.77
Elective Surgery[d]	944 (44.4)	306 (32.8)	<0.001
Operative Stress Score[e]			
Low (1-2)	330 (16.4)	168 (18.8)	<0.001
Moderate (3)	967 (48.1)	511 (57.1)	
High (4-5)	715 (35.5)	216 (24.1)	

[a] p-value for age corresponds to a chi-square test, as age was analyzed by category (the database censors ages for patients age ≥ 90)
[b] "Other" race category includes Asian, Native Hawaiian or Pacific Islander, and American Indian or Alaska Native
[c] n = 3058 for location prior to admission (0.2% missing)
[d] n = 3059 for elective surgery and non-elective surgery (0.2% missing)
[e] n = 2907 for operative stress score low, moderate, and high (5.1% missing)

Abbreviations: mFI-5 = 5-Factor Modified Frailty Index; COPD = chronic obstructive pulmonary disease; ASA = American Society of Anesthesiologists; SIRS = systemic inflammatory response syndrome

© 2023 Allen MB et al. *JAMA Network Open*.

eTable 2. Univariable and Multivariable Logistic Regression Models Examining the Association Between Frailty and Outcomes Following Perioperative Cardiopulmonary Resuscitation[a]

Outcome	n events/n at risk		Odds Ratio (95% CI)	*P*	Adjusted Odds Ratio[b] (95% CI)	*P*
	mFI-5 < 2	mFI-5 ≥ 2				
30-day Mortality	1,208/2,130	589/934	1.30 (1.1, 1.53)[c]	0.001	1.09 (0.90, 1.31)[c]	0.37
Non-home Discharge	291/867	158/297	2.25 (1.72, 2.94)[c]	<0.001	1.50 (1.10, 2.04)[c]	0.01

[a] Perioperative arrests defined as arrests occurring intraoperatively or postoperatively on the day of surgery (i.e., postoperative day 0)

[b] Adjusted for age, sex, American Society of Anesthesiologists physical status, race, emergency surgery, and sepsis

[c] Ratio of patients with 5-factor Modified Frailty Index ≥ 2 vs < 2

Abbreviations: mFI-5 = 5-factor Modified Frailty Index

© 2023 Allen MB et al. *JAMA Network Open*.
