# Supplementary material for: Frailty and Outcomes Following Cardiopulmonary Resuscitation for Perioperative Cardiac Arrest
Source: JAMA Netw Open. 2023 Jul 3;6(7):e2321465. doi: 10.1001/jamanetworkopen.2023.21465 (PMC10318473; doi:10.1001/jamanetworkopen.2023.21465)
Supplement: Supplement 2. — Data Sharing Statement [file jamanetwopen-e2321465-s002.pdf]

Data Sharing Statement

Allen. Frailty and Outcomes Following Cardiopulmonary Resuscitation for Perioperative Cardiac Arrest. *JAMA Netw Open*. Published July 03, 2023.
doi:10.1001/jamanetworkopen.2023.21465

Data
Data available: No

Additional Information
Explanation for why data not available: The data are available through ACS-NSQIP.
